# COVID-19 collaborative modelling for policy response in the Philippines, Malaysia and Vietnam

**DOI:** 10.1016/j.lanwpc.2022.100563

**Published:** 2022-08-11

**Authors:** Angus Hughes, Romain Ragonnet, Pavithra Jayasundara, Hoang-Anh Ngo, Elvira de Lara-Tuprio, Maria Regina Justina Estuar, Timothy Robin Teng, Law Kian Boon, Kalaiarasu M. Peariasamy, Zhuo-Lin Chong, Izzuna Mudla M Ghazali, Greg J. Fox, Thu-Anh Nguyen, Linh-Vi Le, Milinda Abayawardana, David Shipman, Emma S. McBryde, Michael T. Meehan, Jamie M. Caldwell, James M. Trauer

**Affiliations:** a School of Public Health and Preventive Medicine, Monash University, Melbourne Australia; b Woolcock Institute of Medical Research, Hanoi, Viet Nam; c Usher Institute, The University of Edinburgh, Edinburgh, United Kingdom; d Department of Mathematics, Ateneo de Manila University, Manila, Philippines; e Department of Information Systems and Computer Science, Ateneo de Manila University, Manila, Philippines; f Institute for Clinical Research, National Institutes of Health, Ministry of Health Malaysia, Kuala Lumpur, Malaysia; g Institute for Public Health, National Institutes of Health, Ministry of Health Malaysia, Kuala Lumpur, Malaysia; h Malaysian Health Technology Assessment Section, Medical Development Division, Ministry of Health Malaysia, Kuala Lumpur, Malaysia; i Central Clinical School, Faculty of Medicine and Health, The University of Sydney, Sydney, Australia; j WHO Regional Office for the Western Pacific, Manila, Philippines; k Australian Institute of Tropical Health and Medicine, James Cook University, Townsville, Australia; l High Meadows Environmental Institute, Princeton University, New Jersey, United States of America

**Keywords:** Modelling, COVID-19, Policy, Western Pacific

## Introduction

Mathematical models that capture COVID-19 dynamics have supported public health responses and policy development since the beginning of the pandemic,^1^, ^2^ yet there is limited discourse to describe features of an optimal modelling platform to support policy decisions or how modellers and policy makers have engaged with each other. Here, we outline how we used a modelling software platform to support public health decision making for the COVID-19 response in the Western Pacific Region (WPR) countries of the Philippines, Malaysia and Viet Nam. This perspective describes an approach to support evidence-based public health decisions and policy, which may help inform other responses to similar outbreak events. The platform we describe formed the basis for one of the inaugural World Health Organization (WHO) Western Pacific (WPRO) Innovation Challenge awards, and was backed by collaboration between epidemiological modellers, those providing public health advice, and policy makers.

## The epidemiological context

**Figure 1:**
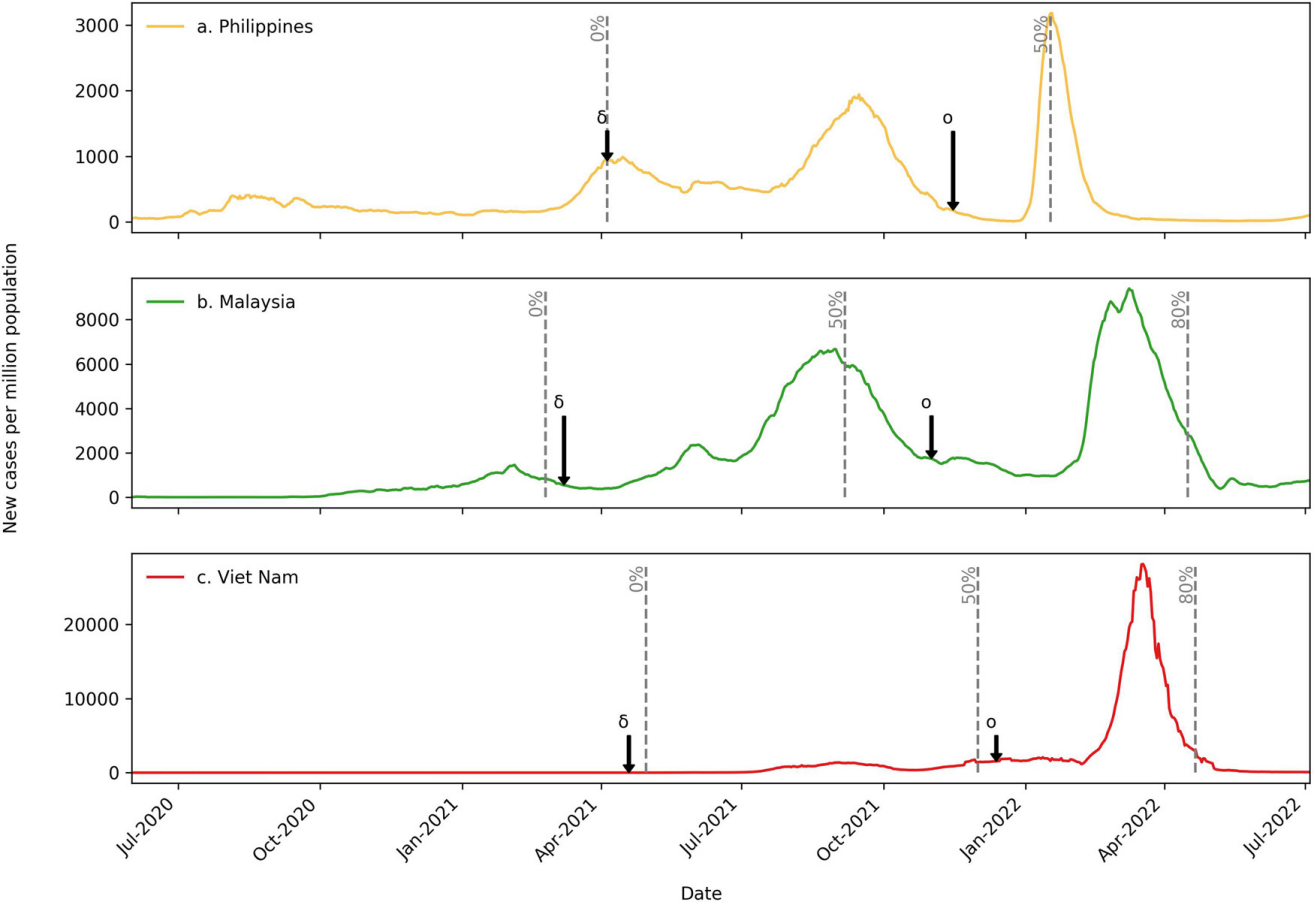
Epidemic curves over the course of the pandemic for a. the Philippines, b. Malaysia and c. Viet Nam. Curves show the 7-day moving average of new cases per million population as of 4 July 2022. Daily reported case data for each country sourced from the COVID-19 data repositry made available by the Centre for Systems Science and Engineering at John Hopkins University.^5^ Note the difference in y-axis scales. Grey dashed lines indicate vaccination coverage milestones. δ = Date of first day of fortnightly reporting for first Delta case in each country by covariants.org. o = Date of first day of fortnightly reporting for first Omicron case in each country by Covariants.org. Covariants.org utilises genomic sequences shared via GISAID, the global data science initiative.^6^, ^7^ Start = commencement of vaccine rollout in the general population (estimated as the first date that population coverage was reported), 50% = 50% 2-dose coverage and 80% = 80% 2-dose coverage. 80% coverage not shown for Philippines, as 2-dose coverage less than 80% as of 4 July 2022.All estimates use whole population estimates as the denominator to facilitate comparison between countries and avoid issues with policy changes in age-specific vaccine eligibility over time. Vaccine coverage data sourced from Data on COVID-19 vaccinations, made available by Our World in Data.^10^

The WPR includes demographically, socioeconomically and culturally diverse countries, which has resulted in major differences in the timing, size, and duration of COVID-19 epidemic waves.^3^ Variations in public health responses and differential access to vaccine supply have further contributed to these differences.^4^ Figure 1 illustrates the different trajectories of the lower-middle income countries (LMIC) the Philippines and Viet Nam, and the upper-middle income country Malaysia over the course of the pandemic.^5^, ^6^, ^7^ This demonstrates that early in the pandemic, the Philippines, Malaysia and Viet Nam, were able to maintain a low COVID-19 burden compared to countries in highly affected regions, such as Europe and the Americas.^4^ This was achieved through various combinations of international border restrictions with quarantine for incoming travellers, lockdowns (“community quarantine” or “movement control” orders), personal protective behaviours (e.g., mask wearing and social distancing), and strong testing and isolation strategies.^8^ However, like many countries around the world, they have experienced a high burden of transmission and infection following the emergence of the Delta and Omicron variants (Figure 1, Table 1).^3^^,^^9^

**Table 1:** Summary of modelling initiatives by country.

	*Country application*		
	Philippines	Malaysia	Viet Nam
***Key country stakeholders and liaison organisations***			
*National agencies*	The Sub-Technical Working Group on Data Analytics of the Inter-Agency Task Force for Emerging Infectious Diseases (IATF STWG DA) Epidemiology Bureau, The Department of Health	The Malaysian Health Technology Assessment Section, Ministry of Health (MaHTAS) Institute for Clinical Research (ICR), NIH Institute for Public Health (IKU), NIH	The Ministry of Information and Communications
*University-affiliated research groups*	Ateneo de Manila University Monash University James Cook University	Monash University	Monash University The University of Sydney
*International agencies*	WHO Country Office WHO Regional Office for the Western Pacific	WHO Country Office WHO Regional Office for the Western Pacific	The Australian Department of Foreign Affairs and Trade
*Independent not-for-profit research institutes/groups*	FASSSTER^a^		The Woolcock Institute of Medical Research, Viet Nam
***COVID-19 burden***^b^			
*Cumulative cases (per million)*	32,573	136,291	110,286
*Cumulative deaths (per million)*	532	1066	442
***Vaccination***^c^			
*2-dose coverage*	62.8%	81.4%	82.3%
*Booster coverage*	13.4%	48.6%	63.0%
***Example policy questions***			
*Disease burden and healthcare capacity*	• What is the projected size and peak in COVID-19 cases, hospitalisations, ICU beds occupied, and deaths from the Delta variant in Calabarzon, July to September 2021? • What is the projected size and peak in COVID-19 cases, hospitalisations, ICU beds occupied, and deaths from the Omicron variant in the National Capital Region, Philippines, January to February 2022?	• What is the projected size and peak in COVID-19 cases, hospitalisations, ICU beds occupied, and deaths at a national level from the Delta variant, August to October 2021? • What are the projected effects on COVID-19 case numbers and health care utilisation at a national level following the introduction of new variants of concern with particular characteristics into Malaysia following the Omicron wave in February to March 2022?	• What is the projected size and peak of COVID-19 cases, hospitalisations, ICU bed occupancy, and fatalities from the Delta variant in Ho Chi Minh City, July to December 2021? • What is the projected size and peak of COVID-19 cases, hospitalisations, ICU bed occupancy, and fatalities from the Omicron variant in Hanoi, February to April 2022?
*Reducing COVID-19 transmission*	• How can compliance to minimum public health standards (MPHS) help to reduce transmission? • What are the projected effects of public health interventions on the Delta wave trajectory? What local community quarantine policy will optimally mitigate infection spread? • What are the projected effects of school openings and easing of mobility restrictions on the Omicron epidemic?	• Vaccination risk-benefit analysis: Comparison of the projected number of hospitalisations and deaths prevented due to immunisation • What are the projected effects of school reopenings and easing of mobility restrictions on the Delta wave epidemic?	• What are the projected effects of different combinations of school and border reopenings, and easing of mobility restrictions on the Delta and Omicron epidemics? • Vaccination cost-benefit analysis: what is the optimal booster interval for the spring 2021 vaccination campaign? • What are optimal vaccination recommendations for people who have completed the two-dose scheme?
***Output format***			
	Model projections provided to in-country modellers for incorporation in presentations of the sub-technical working group on Data Analytics (sTWG-DA) to IATF principals	Policy briefs and modelling projection with different scenarios to Ministry of Health and the WHO Country Office	Public media releases and policy briefs to the Office of Government, the Ministry of Health, and the COVID-19 Policy Advisory and Economic Recovery Group for Ho Chi Minh City
***Publications***			
	Understanding COVID-19 dynamics and the effect of interventions in the Philippines: A mathematical modelling study. Caldwell et al. (2021)	Sustaining effective COVID-19 control in Malaysia through large-scale vaccination. Jayasundara et al. (2021)	Draft manuscript only.

a See link: DOH: FASSSTER COVID-19 v4.0 (ehealth.ph) for explanation of FASSSTER.

b Cumulative cases and deaths as of 4th July 2022. Daily reported case and death data for each country sourced from the COVID-19 data repositry made available by the Centre for Systems Science and Engineering at John Hopkins University.^6^

c 2-dose and booster dose coverage as of July 7 2022. Vaccine coverage data sourced from Data on COVID-19 vaccinations, made available by Our World in Data.^10^

These differences in epidemiology highlight the importance of delivering context-dependent modelling to support local public health decision-making. To capture transmission and disease dynamics accurately, modelling must be relevant to the current local context with respect to surveillance data, population demographics, underlying population immunity, and the implementation and adherence to public health and social measures over time (PHSM, such as facemasks and mobility restrictions).

## A software platform to support infectious disease policy decisions

**Figure 2:**
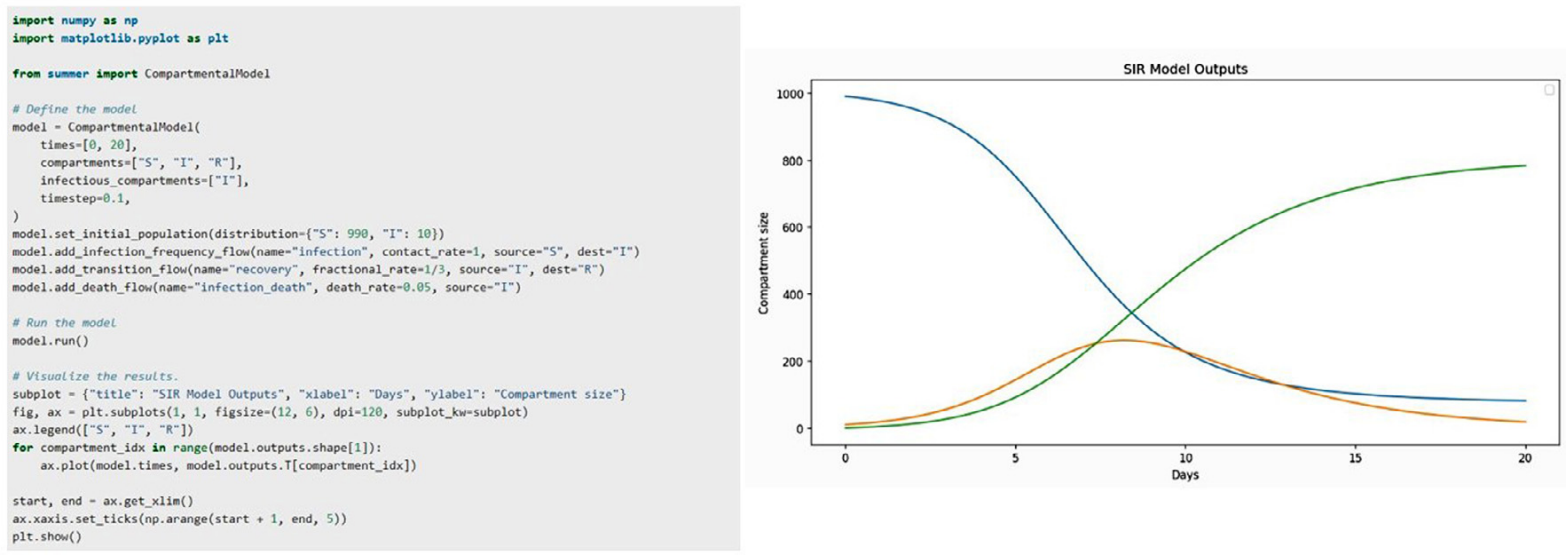
Illustration of code and simple visualisation of construction of a basic Susceptible-Infected-Exposed model using the Summer package, which provides a domain-specific syntax for infectious disease modelling. Summer underpins model construction using the AuTuMN platform (Summer documentation (summerepi.com)). Although actual models are typically more complex, the underlying principle with which we construct compartment models is demonstrated.

The open-access modelling software platform “AuTuMN” served as a key tool to capture COVID-19 dynamics in the Philippines, Malaysia, and Viet Nam.^11^ The aim of our platform is the integration of epidemiological concepts and dynamic disease transmission models with software engineering and data science principles. In Figure 2, we show an example of the code needed to construct a simple susceptible-infected-exposed (SIR) model using features of the platform. The platform's central infrastructure is designed around pluggable modules tailored to specific epidemiological phenomena and is applicable to a range of contexts. In addition to features relevant to infectious diseases, the infrastructure allows for deployment to cloud computing environments and integration with commonly used data science tools, such as Jupyter notebooks. This includes functionality for retrieving and interacting with model outputs locally, such that the platform aims to optimise both computational performance and end-to-end modelling workflow. The platform was designed and developed with open-source software best practices in mind.^12^ This approach has many benefits, but the key principles are transparency, reproducibility, error avoidance, and collaboration through online version control utilising the widely used source code management platform Github (https://github.com/monash-emu/AuTuMN). This practice is in line with a growing movement to ensure that the code used to produce COVID-19 models and projections is shared openly.^13^^,^^14^ Results and analysis of specific modelling activities were rapidly communicated through online, interactive dashboards, which policy makers could use to query and investigate scenarios directly.

## Influencing policy

### Overview

**Figure 3:**
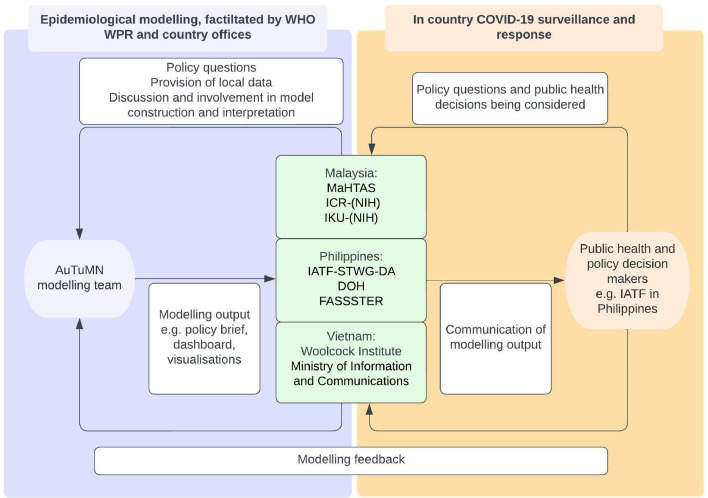
Description of workflow for modelling collaboration indicating; key collaborators, sharing of data and information and feedback. IATF STWG DA = The Sub-Technical Working Group on Data Analytics of the Inter-Agency Task Force for Emerging Infectious Diseases, DOH = Department of Health, MaHTAS = The Malaysian Health Technology Assessment Section, Ministry of Health, ICR-(NIH) = Institute for Clinical Research, National Institute of Health, IKU-(NIH) = Institute for Public Health, National Institute of Health.

**Figure 4:**
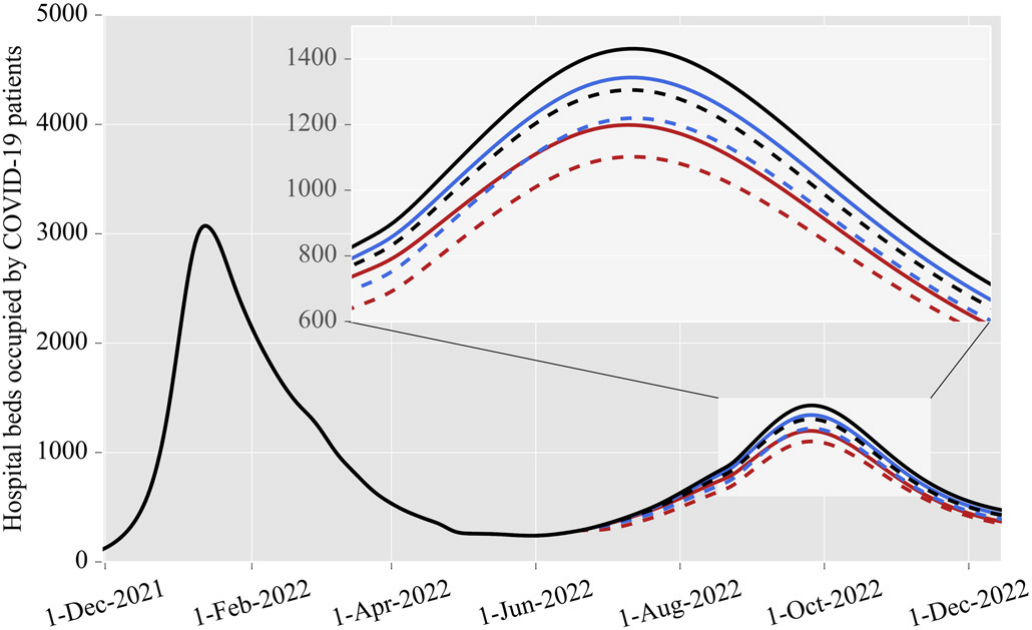
Example of modelling projections to support policy decisions. Figure shows projected hospital occupancy in the Philippines National Capital Region under various booster vaccination scenarios. We consider monthly booster vaccination rates of 200,000 (black lines), 500,000 (blue lines) and 1,000,000 (red lines). Scenarios assuming uniform booster allocation across all age groups are shown with solid lines. Scenarios where older individuals are prioritised for booster vaccination are shown with dashed lines.

The engagement we describe here was instigated to provide near real-time, mechanistic models that simulated transmission dynamics for viral spread in the Philippines, Malaysia, and Viet Nam to assist in answering policy-relevant questions for COVID-19 control. Table 1 outlines the modelling initiative in each country, highlighting the specific collaborating groups, key policy questions modelled, and how the results were communicated. An overview of the general workflow is presented in Figure 3 and an example application of the platform to a policy question is presented in Figure 4. In this example, we highlighted how various booster vaccination strategies could affect the size of a future epidemic wave due to waning of infection-induced immunity.

### Building collaborative teams

WHO WPR regional and country offices, along with the Woolcock Institute of Medical Research, played the important role of facilitating collaboration among the modelling team and in-country collaborators. This collaborative network included national departments/ministries of health, independent not-for-profit research institutes, government-supported research institutes, and university-affiliated research groups, that included some local in-country modelling and surveillance expertise (Table 1).

Trust developed between in-country staff (Figure 3) and the modelling team, supporting an open forum for the discussion and critique of important knowledge for the development and interpretation of dynamic transmission models. Country teams involved in the local COVID-19 response contributed vital situational awareness of current epidemiology and the policy and public health decisions under consideration. When issues arose, appropriate points of contact were established, allowing for quick trouble-shooting. For instance in the Philippines, early in the pandemic, the modelling team noticed unusual patterns of reported COVID-19 mortality, and local public health surveillance expertise was able to identify the patterns being due difficulties in classifying COVID-19 as a cause of death and other reasons delaying reporting. Similarly, when in-country teams noticed modeling patterns that did not reflect local intuition, the reasons for any apparent discrepancies could be identified, and revision to the modelling approach considered. Once all partners were satisfied with the model outputs, in-country teams could then use the co-developed modelling results in the policy and decision-making process. This allowed teams working in COVID-19 surveillance and response to integrate modelling within the broader disease surveillance context.

### Acceptance and skepticism

Building trust in modelling amongst public health and policy decision-makers working on pandemic response takes time and can be met with a range of responses. Models that capture the heterogeneities of the COVID-19 epidemic and are able to address the specific policy questions (e.g., age-specific vaccine allocation) often require substantial complexity. At the same time, stakeholders in national pandemic responses may have limited experience in the use or interpretation of modelling. To counter these limitations, we sought to provide regular explanations of the model components and associated reasons for the models’ findings to different stakeholder groups and compare the findings with results from complementary models where possible. The goal was to equip public health officials and decision makers with the ability to critically assess the modelling results and understand the inherent strengths and limitations in what should be inferred.

### Obstacles for costly interventions

Globally, policy makers were forced to make decisions about how to manage COVID-19 given incomplete and often conflicting information, considering the pros and cons of measures of PHSMs. The aim of our work was to provide policy makers with regular updates on the most relevant and realistic scientific evidence given local context and concerns. We showed the expected impact of different decisions on public health (e.g., projected cases, hospitalisations) to support decision making, but generally did not take our own stance or negotiate for specific policy decisions, as our teams did not include experts in other sectors (e.g., economists, educators, industry) relevant to final policy decisions.

## Measuring impact

Impacts of the modelling we describe may include: (i) strengthening international collaboration and capacity among infectious disease and pandemic stakeholders in the WPR; (ii) contributing to scientific knowledge about COVID-19 dynamics in LMIC and epidemiological modelling; and (iii) exposure of our software platform to a diverse range of epidemiological settings and questions.

Partners involved in this work have gained new knowledge, skills, and colleagues as a result of this process. From a policy standpoint, we recognize that modelling is only useful to policy decisions when it is both correct and can inform actionable decisions. Public health interventions are complex, and require decision makers to synthesize multiple lines of evidence, of which modelling is just one component. Some examples of where the modelling presented here contributed to public health action include: accurately predicting the timing and magnitude of epidemic peaks to help inform health system capacity planning, identifying epidemic resurgence was likely due to the emergence of a new variant of concern in the Philippines and demonstrating how differences vaccination coverage and delivery rate in Malaysia may affect health system capacity requirements. A reflection of this was the acknowledgement of modelling outputs in public health media releases in Viet Nam and the Philippines.^15^^,^^16^

From a scientific standpoint, the collaboration has so far led to the production of two peer-reviewed papers in the country contexts of the Philippines and Malaysia,^17^^,^^18^ involving multiple members from across the range of partners and sectors involved. This highlights the strength of the collaboration and the trust that was built between modellers and in-country stakeholders. The publications were important in bringing the modelling into the public domain to support transparency and public engagement.^17^ Finally, this process has led to investment in, refinement, and testing of the AuTuMN platform, expanding its applicability to more epidemiological contexts and questions

## Lessons learnt

### Cross-sectoral collaboration enhances model development

We found that cross-sectoral collaboration and model co-development with local experts in each country was crucial to the modelling-policy cycle, confirming previous findings.^19^^,^^20^ This supported access to and understanding of up-to-date and relevant information regarding: i) epidemiological and healthcare data, ii) the public health policy and interventions being considered, iii) the generation of transmission models, and 4) the social and epidemiological context and interpretation of model simulations. Examples included knowledge of holiday periods, typical timing of peak transmission of respiratory viruses, timing of restriction changes such as schools reopening and local observations of behavioural changes such as reductions in social distancing and mask wearing. Having this valuable local knowledge and context was in turn beneficial to in-country collaborators, as it allowed for model scenarios to reflect real-world public health decisions and models to estimate epidemiological parameters with uncertainty within the local context (e.g., the relative transmissibility of variants).

A novel, beneficial component to the collaborations in each country has been the involvement of in-country modellers such as those from FASSSTER and Ateneo de Manila University in the Philippines, the National Institute of Health (NIH) in Malaysia and the Woolcock institute, Viet Nam (Table 1). This assisted in the clear communication and understanding of the results and underlying assumptions of our models to in-country decision makers and in some cases permitted some comparison to different models.

### Challenges

The need for rapid evidence-based projections for a newly emerging and constantly changing disease presented major challenges. With many of the AuTuMN modellers coming from a background in tuberculosis modelling, for which the cycle of modelling for policy can last several months, the pressures of pandemic modelling necessitated a major shift in approach. Results were often required within days, and within a few weeks projections could become irrelevant due to changing epidemiology and policy priorities. This compounded the challenges associated with exploring and communicating model assumptions and uncertainty in outputs to be relevant to policy makers.^2^ While model parameter choices can be explored through standard calibration algorithms, the full uncertainty in model projections should ideally incorporate structural choices in model construction, which were difficult to explore fully under the time constraints of the pandemic policy cycle. Further, while our platform supports the rapid addition of model structures to capture processes relevant to specific policy questions, in practice there is still a considerable need for user input. For example, structure that captures multiple competing strains can be rapidly added through our platform, but presents issues with calibration and output interpretation that still place a burden on the human operator.

### Future directions

Changes in factors such as variants, disease dynamics, and vaccine coverage is leading to a shift in COVID-19 public health response policy. Moving forward, developing a sustainable and adaptable public health response to COVID-19 will be essential for countries in the region to mitigate the effects of the disease. Infectious disease modelling should be progressively integrated into domestic response capacity in LMICs of the WPR for pandemic preparedness and infectious diseases more broadly. With this in mind, we are now further expanding our platform functionality and moving towards a capacity development approach for the Philippines, Malaysia and Viet Nam.

## Contributors

**A.H.:** Conceptualisation, Visualisation, Writing – Original Draft, Writing – Review & Editing. **P.J.:** Conceptualisation, Writing – Review & Editing. **H.A.N.:** Conceptualisation, Writing – Review & Editing. **R.R.:** Conceptualisation, Writing – Review & Editing. **E.L.T.:** Conceptualisation, Writing – Review & Editing. **M.R.J.E.:** Conceptualisation, Writing – Review & Editing. **T.R.T.:** Conceptualisation, Writing – Review & Editing. **L.K.B.:** Conceptualisation, Writing – Review & Editing. **K.M.P.:** Conceptualisation, Writing – Review & Editing. **Z.L.C.:** Conceptualisation, Writing – Review & Editing. **I.M.M.G.:** Conceptualisation, Writing – Review & Editing **G.J.F.:** Conceptualisation, Writing – Review & Editing. **T.A.N.:** Conceptualisation, Writing – Review & Editing. **L.V.L.:** Conceptualisation, Writing – Review & Editing. **M.A.:** Conceptualisation, Writing – Review & Editing. **D.S.:** Conceptualisation, Writing – Review & Editing. **E.S.M.:** Writing – Review & Editing **M.T.M.:** Conceptualisation, Writing – Review & Editing **J.M.C.:** Conceptualisation, Writing – Review & Editing and **J.M.T.:** Conceptualisation, Writing – Original Draft, Writing – Review & Editing, Supervision.

## Declaration of interests

The authors declare no conflicts of interest. The modelling work was supported by the World Health Organization Regional Office for the Western Pacific to provide modelling advice to Member States. Authors HAN and TAN were supported by funding from the Department of Foreign Affairs and Trade (DFAT), Australia.

## References

[1] McBryde ES, Meehan MT, Adegboye OA (2020). Role of modelling in COVID-19 policy development. Paediatr Respir Rev.

[2] Holmdahl I, Buckee C. (2020). Wrong but useful — what covid-19 epidemiologic models can and cannot tell us. N Engl J Med.

[3] Organization WH (2022).

[4] Chen Y-Y, Assefa Y. (2021). The heterogeneity of the COVID-19 pandemic and national responses: an explanatory mixed-methods study. BMC Public Health.

[5] Dong E, Du H, Gardner L. (2020). An interactive web-based dashboard to track COVID-19 in real time. Lancet Infect Dis.

[6] Shruti K, Céline G, Lucas F (2021). GISAID's role in pandemic response. China CDC Weekly.

[7] Hodcroft EB. (2021). https://covariants.org/.

[8] Patel J, Sridhar D. (2020). We should learn from the Asia-Pacific responses to COVID-19. Lancet Reg Health West Pac.

[9] Kwok KO, Huang Y, Tsoi MTF (2021). Epidemiology, clinical spectrum, viral kinetics and impact of COVID-19 in the Asia-Pacific region. Respirol.

[10] Mathieu E, Ritchie H, Oritz-Ospina E (2021). A global database of COVID-19 vaccinations. Nat Hum Behav.

[11] Trauer JM, Ragonnet R, Doan TN, McBryde ES. (2017). Modular programming for tuberculosis control, the “AuTuMN” platform. BMC Infect Dis.

[12] Hunter-Zinck H, de Siqueira AF, Vásquez VN, Barnes R, Martinez CC. (2021). Ten simple rules on writing clean and reliable open-source scientific software. PLoS Comput Biol.

[13] Barton CM, Alberti M, Ames D (2020). Call for transparency of COVID-19 models. Science.

[14] Jalali MS, DiGennaro C, Sridhar D. (2020). Transparency assessment of COVID-19 models. Lancet Glob Health.

[15] Manh Hoa,Tanh An. Secretary of the Ho Chi Minh City Party Commitee Nguyen Van Nen: Adapting is not enough, it is necessary to actively construct and create. Sai Gon Gai Phong - SGGP Online. 2021 [cited 2022 Jul 1]. Available from: https://www.sggp.org.vn/bi-thu-thanh-uy-tphcm-nguyen-van-nen-thich-ung-la-chua-du-can-chu-dong-kien-tao-va-sang-tao-782195.html.

[16] Republic of the Philippines, Department of Health. Manilla: DOH; 2022. Continuous decline in MPHS compliance could lead to as high as half a million active COVID-19 cases. [cited 2022 Jul 1]; [about 1 screen]. Available from: https://doh.gov.ph/press-release/DOH-CONTINUOUS-DECLINE-IN-MPHS-COMPLIANCE-COULD-LEAD-TO-AS-HIGH-AS-HALF-A-MILLION-ACTIVE-COVID-19-CASES.

[17] Caldwell JM, de Lara-Tuprio E, Teng TR (2021). Understanding COVID-19 dynamics and the effects of interventions in the Philippines: a mathematical modelling study. Lancet Reg Health.

[18] Jayasundara P, Peariasamy KM, Law KB (2021). Sustaining effective COVID-19 control in Malaysia through large-scale vaccination. Epidemics.

[19] Teerawattananon Y, KC S, Chi YL (2022). Recalibrating the notion of modelling for policymaking during pandemics. Epidemics.

[20] Aguas R, White L, Hupert N (2020). Modelling the COVID-19 pandemic in context: an international participatory approach. BMJ Glob Health.

